# Editorial: What Is Musical Creativity? Interdisciplinary Dialogues and Approaches

**DOI:** 10.3389/fpsyg.2021.796096

**Published:** 2021-11-22

**Authors:** Andrea Schiavio, David Michael Bashwiner, Rex Eugene Jung

**Affiliations:** ^1^Centre for Systematic Musicology, University of Graz, Graz, Austria; ^2^Department of Music, University of New Mexico, Albuquerque, NM, United States; ^3^Department of Psychology, University of New Mexico, Albuquerque, NM, United States

**Keywords:** musical creativity, music cognition, music neuroscience, music performance, creative cognition

## Introduction

Creativity is central to human life, and the domain of music is no exception (Boden, 2004; Cook, 2018). From learning to play an instrument to performing, composing, and improvising, much of our music-making activities are deeply associated with creative thought and action (van der Schyff et al., 2018). Given its complex phenomenology and variety of manifestations, understanding musical creativity remains a crucial, yet difficult goal of current scholarship on the musical mind. How exactly can musical creativity be defined? What are its main characteristics, and how do these play out across different musical settings? On what neural, social, cognitive, and behavioral resources it is based? We are convinced that clarifying what musical creativity entails requires a dialogue between theoretical analysis, experimental research, and the practical teaching of everyday music-making. To do so, we have deliberately invited submissions from colleagues working in diverse areas, promoting a cross-pollination of ideas and insights. Hence, this edited collection includes articles exploring how creativity plays out in concrete musical contexts from a range of perspectives: here the views of composers, music theorists, musicologists, neuroscientists, ethnomusicologists, educators, and psychologists, take the form of conceptual analyses, literature reviews, and original empirical studies, ensuring a complementarity of epistemological approaches and methods. Such a variety of contributions fosters fascinating opportunities to examine from new angles the mechanisms associated with creative practice and experience, as well as their interplay with broader aspects of human cognition. This allows us to explore, in novel ways, the neural, psychological, and behavioral processes involved in (the development of) musical creativity; to gain a deeper understanding of the social and individual dimensions of creative music-making; to put novel ideas and hypotheses to the test; and to offer syntheses of methodologies and findings pertaining to diverse research domains. While it is neither possible nor necessary to reduce the findings of the contributions in this Research Topic to a discrete number of outcomes, nevertheless the approaches taken can be grouped into the following three categories: (i) reviews and theoretical investigations, where existing assumptions and conceptual frameworks are systematically re-examined on the basis of novel insights, (ii) contextual framings, in which musical creativity is addressed within specific domains of interest (e.g., music performance), and (iii) implementations in composition and musical analysis, where novel theoretical and practical tools are proposed to illuminate how creative thought develops across a web of compositional processes. In what follows, we describe how the content of each contribution speaks to one of these categories, and we offer general insights intended to stimulate further discussion across diverse areas of enquiry and practice.

## Reviews and Theoretical Investigations

Contributions pertaining to the category “reviews and theoretical investigations” offer innovative approaches to reconceptualizing existing findings and systematically reexamining previous theoretical assumptions. An excellent example of such recontextualization can be found in the article by Barrett et al., which challenges past biases in the scientific literature privileging individualist conceptualizations of creative production over more collaborative conceptualizations. Conducting a systematic review of recent literature on collaborative musical creativity, the authors examined factors such as the research setting (educational or professional), the style of music featured (jazz being the most common), the main questions asked and reasons for asking them (focusing on learning vs. on the artistic product), and how social factors and musical factors were seen to interact. Among other things, the researchers report that the vast majority of the contributions considered were found to rely on qualitative methods, and that all but one had addressed only Western styles of music. Future studies seeking to correct these imbalances might therefore aim to offer more diversity regarding methodological approach and cultural setting. By providing greater conceptual clarity and broader views, allowing findings to be compared and generalized, such future studies might in turn inspire novel approaches to examining and challenging pre-existing theoretical assumptions and biases, of which we may be largely unaware.

The fascinating article by Schubert goes in this direction when it asks us to rethink existing definitions of creativity, proposing instead a new framework based on a *spreading activation model*. On this view, creative (e.g., musical) ideas might be best understood as highly connected nodes which “encode, store, process, and recall simple pieces of information.” By exploring the principles governing the network in which such nodes operate, the role of positive affect for creative activity is given major emphasis.

A complementary analysis on the interactive and action-based components of musical creativity is offered by Schiavio and Benedek. Drawing on the conceptual resources of enactive cognitive science (see Varela et al., 1991), they examine creative cognition as an adaptive phenomenon that “originates in a primordial, and necessary, sense-making activity—a bio-cognitive inclination to create, transform, and maintain viable relationships with the world.” The move, it is suggested, can help mitigate two of the most important dichotomies of the field—that between individuality and collectivity and that between domain-generality and domain-specificity.

Exploring the biological and adaptive roots of creative cognition is also the main goal of the article by Podlipniak, which examines human musical creativity from the perspective of gene-culture coevolution. According to this perspective, creative behaviors have downstream effects upon gene flow, and gene flow in turn feeds back to influence those behaviors. Two opposing forces are proposed as central to such gene-behavior interaction: plasticity and canalization. Behaviors must be sufficiently plastic to be differently acted upon by natural and sexual selection. However, too much variance can be invisible to selection, and hence only behaviors that remain consistent over time, becoming canalized, sufficiently influence genetic transmission. The inexhaustible creative potential of the human musical system is proposed to arise not solely from plastic forces, but from the interaction of such forces with canalized structures such as the hierarchies of pitch and rhythm found across virtually all human cultures.

An approach to musical creativity based on statistical learning is developed by Daikoku et al.. Having reviewed important research on the neural and computational roots of statistical creativity, they propose a hierarchical model that brings together *shallow* and *deep* statistical learning (see e.g., LeCun et al., 2015), suggesting that musical creativity involves the integration of shareable units of information and the temporal dynamics of uncertainty. As we will see next, a similar focus on brain dynamics remains a core aspect of research on musical creativity, particularly when it seeks to address concrete questions contextually.

## Contextual Framings

Musical creativity is expressed through a variety of manifestations, processes, and outcomes spanning a range of situated contexts and dimensions. The contributions that fall under the category “contextual framings” offer an in-depth look into one or more of these dimensions. The article by Farrugia et al., for example, reports on a single-subject, EEG study based on an ecological paradigm of live musical improvisation. In the experiment, electroencephalography was combined with retrospective ratings to allow a “mental replay” of the variety of subjective states involved in the performance, with a particular focus on the temporal dimension (i.e., the internal time felt by the improviser) characterizing the creative activity.

The musical brain is also at the heart of the contribution by Colombo et al.. Here, two groups of participants were asked to rate the creativity of a musical piece they had just listened to. The first group received transcranial direct current stimulation (tDCS) to inhibit the activation of the auditory Mirror Neuron mechanism (see e.g., Keysers et al., 2003), while the second group served as a control (receiving only sham stimulation). Results showed that, among other things, participants in the first group rated the stimulus as less creative than the second group did, suggesting that the evaluation of specific aspects of musical creativity (i.e., innovation and excitement) partially relies on mirror-like activity.

The context of human-computer co-creation represents another area where various aspects of musical creativity may be explored, variously implemented, and put to a test. In this regard, the paper by Zacharakis et al. introduces a computational melodic harmonization assistant (CHAMALEON), and investigates how expert and novice composers make use of it during a melodic harmonization task. Results indicate that novices found the system more useful than experts, and that interaction with CHAMALEON gave rise to more explorative strategies when compared to harmonizations realized without its support.

In a way complementary to the studies previously described, Alkaei and Küssner take a qualitative approach to investigating creativity in the improvisatory tradition of Arabic *taqsim*. The authors interviewed three Berlin-based, professional oud players of Syrian origin about such aspects of the creative process as the difference between improvisation and composition, and how migrating to Europe has influenced their approach to improvisation. This approach appears well-suited to help reveal the richness of subjective creative experience from a transcultural perspective, helping to build a new picture of how *taqsim* is understood and performed in relation to an artist's individual agency and culture.

This goal of evaluating and expanding our theoretical assumptions about what creativity is, and how it works, is elegantly pursued by Huovinen. Here the author notes that individual researchers rarely offer explicit arguments for choosing one theory over another, typically proceeding “as if they would have already made up their minds.” Huovinen's solution to the problem is novel: while specialists in the field of creativity may have too much prior experience to offer unbiased comparisons of different theories, students are actually quite capable of “relat[ing] to more complex, scholarly theories,” serving as a valuable step toward assessing theories rather than simply assuming them to be correct *a priori*. Asking a cohort of music students to rate different theories of creativity for their applicability to four types of target activity—composition, improvisation, performance, and ideation— Huovinen finds that students' evaluations and argumentative strategies differed for each realm of activity, as well as differing as a function of the student's musical background.

## Implementations in Composition and Musical Analysis

While, in principle, scientific studies of musical creativity ought naturally to be of interest not only to other scientists but also to the sorts of creative musicians whose behavioral processes are being studied, the conceptual gap between these two realms is not always intuitively easy to bridge. In that light, two contributions in the present Research Topic are likely to be of particular interest for the focus they place on elucidating high-level compositional processes.

The paper by Besada et al. focuses on a single work, Iannis Xenakis's *Psappha*, addressing a specific compositional procedure that is idiosyncratic not only to that composer, but to that specific work. Taking the perspective that a continued belief in “the romantic myth of the lone genius” —portraying a composer's thought processes as *different in kind* from those of “normal” human beings—makes “musical creativity unnecessarily hard to study,” the authors present a reconstruction and analysis of Xenakis's thought processes in the composition of *Psappha* by way of a general model of “normal” or “everyday” creativity, the *blending theory* of Fauconnier and Turner (2002). By focusing specifically on the sense of time in this work—using notions that are general to cognition, not specific to music—the authors not only shed new light on the structure of the work and this specific composer's unique and idiosyncratic creative process, but also pioneer new avenues for exploring how musical meanings can be formed and transformed in the compositional process.

With a similar focus on music composition and analysis, the contribution by Spence introduces a model (named *Experimental Composition Improvisation Continua* or ECIC) thought to capture the range of continuities between composition and improvisation often displayed by experimental music. It is argued that its application might help researchers and analysts to trace, isolate, and compare those indeterminate musical properties that might be attributed to the environment in which the performance takes place, improvisational style, as well as the performer's action or inaction.

In conclusion, the present Research Topic addresses a number of crucial issues in creativity studies by focusing on the domain of music. Here, theoretical, empirical, and practice-based insights are developed, examined, and implemented across different settings, presenting novel findings, and conceptual tools that can be relevant to composers, musicians, and researchers from across diverse fields. While even a sizeable collection such as ours can offer only a partial view upon so complex a topic as musical creativity, our intention, as stated in our title, has been to inspire *dialogue*, and promote novel *approaches* to bringing artists and researchers together across fields, disciplines, cultures, and orientations. In a sense, this represents a valuable way forward *per se*, as many researchers in the field will be able to benefit from the rich interdisciplinary resources developed in the present collection, delve into its multiple theoretical, empirical, and practical dimensions, and access insights and conceptual tools from scholarly territories that may often appear too distant from theirs. It should also be noted that such interdisciplinary dialogues permeate music itself, involving performers, composers, listeners, educators, and scholars. Similarly, the study of creativity has a rich and sprawling history, boasting a vast diversity of approaches—which have considerably expanded understanding, challenged assumptions, and inspired new questions. While there undoubtedly remains much to learn about this fascinatingly alluring faculty, we hope to have inspired new dialogues about, and approaches to, the study of musical creativity.

## Author Contributions

All authors listed have made a substantial, direct, and intellectual contribution to the work, and approved it for publication.

## Funding

AS acknowledges the support of the Austrian Science Fund (FWF). This research was funded in whole, or in part, by the Austrian Science Fund (FWF), project number: P 32460.

## Conflict of Interest

The authors declare that the research was conducted in the absence of any commercial or financial relationships that could be construed as a potential conflict of interest.

## Publisher's Note

All claims expressed in this article are solely those of the authors and do not necessarily represent those of their affiliated organizations, or those of the publisher, the editors and the reviewers. Any product that may be evaluated in this article, or claim that may be made by its manufacturer, is not guaranteed or endorsed by the publisher.
